# Characterisation of ictal and interictal states of epilepsy: A system dynamic approach of principal dynamic modes analysis

**DOI:** 10.1371/journal.pone.0191392

**Published:** 2018-01-19

**Authors:** Zabit Hameed, Saqib Saleem, Jawad Mirza, Muhammad Salman Mustafa, Qamar-ul-Islam

**Affiliations:** 1 Department of Electrical Engineering, Institute of Space Technology, Islamabad, Pakistan; 2 Department of Electrical Engineering, COMSATS Institute of Information Technology, Sahiwal, Pakistan; 3 Centre for Translational Physiology, Department of Surgery and Anaesthesia, University of Otago, Wellington, New Zealand; 4 Department of Electrical Engineering, COMSATS Institute of Information Technology, Islamabad, Pakistan; 5 Department of Mechanical Engineering, COMSATS Institute of Information Technology, Sahiwal, Pakistan; University of Modena and Reggio Emilia, ITALY

## Abstract

Epilepsy is a brain disorder characterised by the recurrent and unpredictable interruptions of normal brain function, called epileptic seizures. The present study attempts to derive new diagnostic indices which may delineate between ictal and interictal states of epilepsy. To achieve this, the nonlinear modeling approach of global principal dynamic modes (PDMs) is adopted to examine the functional connectivity of the temporal and frontal lobes with the occipital brain segment using an ensemble of paediatric EEGs having the presence of epileptic seizure. The distinct spectral characteristics of global PDMs are found to be in line with the neural rhythms of brain dynamics. Moreover, we find that the linear trends of associated nonlinear functions (ANFs) associated with the 2nd and 4th global PDMs (representing delta, theta and alpha bands) of Fp1–F3 may differentiate between ictal and interictal states of epilepsy. These findings suggest that global PDMs and their associated ANFs may offer potential utility as diagnostic neural measures for ictal and interictal states of epilepsy.

## 1 Introduction

Epilepsy is the second most common neurological disorder after stroke and affects over 50 million people worldwide [1, 2]. According to the International League Against Epilepsy (ILAE) and the International Bureau for Epilepsy (IBE), epilepsy is “a disorder of the brain characterized by an enduring predisposition to generate at least one epileptic seizure and by the neurobiologic, cognitive, psychological, and social consequences of this condition” [3]. Depending on the foci and intensity of the ictal epileptic activity, persistent seizures might also cause momentary deviations in perception and behavior [4–6], mild degree of convulsions [7, 8], and temporary loss of consciousness [9, 10].

Various computer aided diagnostic techniques [11–13] have been deployed with the aim of automatic detection and prediction of seizures by analysing noninvasive electroencephalogram (EEG) signals. However, most of the previous work [14, 15] undertaken hitherto emphasized only on the individual EEG channels and did not examine the connectivity across different brain regions while exploring complex brain dynamics. Recent studies [16–18] sought an evidence of pathological network disorder following the seizure onset, suggesting to quantify the spatio-temporal functional connectivity of different brain segments to reveal its dynamic behaviour.

Towards this direction, various analytical tools [19, 20] have been employed to test the functional coupling among EEG channels. Examples include wavelet coherence [19], phase synchronization [21] and Granger causality analysis [20]. Most of these techniques have the ability to focus only on the linear interactions among EEG signals. Though some nonlinear models [22–24] have also been developed to examine the causal connectivity of brain segments, yet their pathophysiological interpretation remains daunting. Recently, Cao et al. [25] employed the concept of global principal dynamic modes (PDMs) to examine the characteristics of epilepsy, and reported that nonlinear functions associated with global PDMs may serve as pathophysiologically interpretable tools for ictal and interictal states of epilepsy. Of note, this study was limited by the fact that it analyzed only simulated epileptic EEG data generated by a neural mass model, and did not evaluate the utility of proposed indices on a clinical data.

The present study is aimed to externally validate the use of global PDMs as diagnostic measures to differentiate ictal and interictal states of epilepsy by examining a clinical EEG data set. The functional connectivity of the temporal and frontal lobes with the occipital brain segment is examined with T7–P7 and Fp1–F3 as inputs and P3–O1 as an output (of a putative dual-input output PDM model). We hypothesized that 1) the distinct spectral characteristics of global PDMs signify the neural rhythms (delta, theta, alpha, beta and gamma) of brain functioning, and 2) the (linear) trends of associated nonlinear functions of the 2nd and 4th global PDMs of Fp1–F3 might be sensitive to (ictal and interictal) states of epilepsy, and that these parameters may be used as diagnostic indices to separate ictal and interictal states.

## 2 Data collection and pre-processing

The data analysed in the present study are selected from the CHB-MIT scalp EEG database of physionet [26, 27]. The data were collected at Children’s Hospital Boston, USA and contain continuous EEG recordings of pediatric patients suffering from intractable seizures. This dataset contains numerous types of seizures (clonic, atonic and tonic) where onset and end of each seizure were judged by experts [12, 28], and the corresponding annotations are provided on physionet. The conventional 10–20 lead system was used to record EEG at a sampling rate of 256 Hz with 16-bit resolution. This study involved 23 subjects (5 males, 3–22 years; 16 females, 1.5–19 years) with a total of 198 seizures. Gender of one subject was not reported on physionet, and one female subject was recorded twice with a gap of 1.5 years. The number of seizures and their duration varied across each subject. The onset of each epoch corresponds to the onset of ictal/interictal state. Twenty epochs (ten interictal and ten ictal) from ten subjects were selected to form the training dataset and were used for model training. While the remaining 188 ictal epochs formed the test dataset and were analyzed to validate the estimated markers. For model training, 69-second epochs of one interictal and one ictal activity from each of ten subjects of the training dataset were simultaneously extracted from the T7–P7, Fp1–F3 and P3–O1 channels of the recorded EEGs having the minimum presence of artefacts. These channels were selected knowing that these seizure types can be characterised by the selection of left/right temporal lobes (T7/T8) and extrafocal (O1) channels [29], and a channel with high interdependency to these channels [30]. Additionally, these channels carry minimum effects of the ocular activity [31], and also ensure that the nearby channels might generate spurious results due to the volume conduction effects [31]. For validation purposes, the 188 ictal epochs of all duration of the test dataset were analysed to differentiate from interictal epochs of same duration. The detailed description of the data examined in the present study is provided in S1 and S2 Files.

A second-order low-pass infinite impulse response (IIR) Butterworth filter with a cut-off frequency of 40 Hz is applied to the extracted time series in order to remove high frequency contents. Next, the filtered data is demeaned and detrended to remove the baseline drift and occasional artefacts. Furthermore, Grubb’s test [32] is used to remove the occasional measurement artefacts. The data are analyzed using a custom written software in MATLAB (version R2014b; Mathworks).

## 3 Principal dynamic modes analysis

The modeling approach of global PDMs, proposed and developed by V. Z. Marmarelis [33, 34], represents the compact and efficient representation of a given system by estimating the first and second order Laguerre-Volterra kernels from the input-output data. The mathematical details of this modeling methodology can also be found in [34]. Briefly, it begins with the estimation of first- and second-order (self and cross) Volterra kernels in terms of Laguerre functions using a least-square approach from the given input and output time series [31] i.e.,

$$\begin{array}{cc} O\left(n\right)=k_{0} & +\sum_{m_{1}}^{M}k_{\text{T}}\left(m_{1}\right)T\left(n-m_{1}\right)+\sum_{m_{2}}^{M}k_{\text{F}}\left(m_{2}\right)F\left(n-m_{2}\right)\\ & +\sum_{m_{1}}^{M}\sum_{m_{2}}^{M}k_{\text{TT}}\left(m_{1},m_{2}\right)T\left(n-m_{1}\right)T\left(n-m_{2}\right)\\ & +\sum_{m_{1}}^{M}\sum_{m_{2}}^{M}k_{\text{FF}}\left(m_{1},m_{2}\right)F\left(n-m_{1}\right)F\left(n-m_{2}\right)\\ & +\sum_{m_{1}}^{M}\sum_{m_{2}}^{M}k_{\text{TF}}\left(m_{1},m_{2}\right)T\left(n-m_{1}\right)F\left(n-m_{2}\right)\\ & +\epsilon (n), \end{array}$$ (1)

where *T*, *F* and *O* are T7–P7, Fp1–F3 and P3–O1 respectively, *k*_0_ is a zeroth-order kernel (a constant), {*k*_T_, *k*_F_} are first-order kernels of T7–P7 and Fp1–F3 respectively, {*k*_TT_, *k*_FF_ and *k*_TF_} respectively are second-order self- and cross-kernels describing the intermodulatory interactions between T7–P7 and Fp1–F3 at (*m*_1_,*m*_2_) time lags, and *ϵ*(*n*) represents the errors associated with measurement or modeling at time *n*. *M* is the order of the system memory. In the present study, the same value of *M* is assumed for each series expansion term of Eq 1.

Orthonormal Laguerre functions {*b*_*j*_, *j* = 1,…,*L*} are used to estimate the first- and second-order Volterra kernels [35] given by,

bj(m)=α(m-j)/2(1-α)1/2∑k=0j(-1)k(mk)(jk)αj-k(1-α)k. (2)

Here parameter *α* determines the asymptotic exponential decay rate of the Laguerre function. The resultant first- and second-order kernels are given by,

$$k_{\text{F}}\left(m_{1}\right)=\sum_{j_{1}=1}^{L}\beta_{\text{F}}\left(j_{1}\right)b_{j_{1}}\left(m_{1}\right),$$ (3)

$$k_{\text{T}}\left(m_{2}\right)=\sum_{j_{1}=1}^{L}\beta_{\text{T}}\left(j_{1}\right)b_{j_{1}}\left(m_{2}\right),$$ (4)

$$k_{\text{FF}}\left(m_{1},m_{2}\right)=\sum_{j_{1}=1}^{L}\sum_{j_{2}=1}^{j_{1}}\beta_{\text{FF}}\left(j_{1},j_{2}\right)b_{j_{1}}\left(m_{1}\right)b_{j_{2}}\left(m_{2}\right),$$ (5)

$$k_{\text{TT}}\left(m_{1},m_{2}\right)=\sum_{j_{1}=1}^{L}\sum_{j_{2}=1}^{j_{1}}\beta_{\text{TT}}\left(j_{1},j_{2}\right)b_{j_{1}}\left(m_{1}\right)b_{j_{2}}\left(m_{2}\right),$$ (6)

$$k_{\text{TF}}\left(m_{1},m_{2}\right)=\sum_{j_{1}=1}^{L}\sum_{j_{2}=1}^{j_{1}}\beta_{\text{TF}}\left(j_{1},j_{2}\right)b_{j_{1}}\left(m_{1}\right)b_{j_{2}}\left(m_{2}\right),$$ (7)

where *β* = [*β*_0_ *β*_T_ *β*_TT_ *β*_F_ *β*_FF_ *β*_TF_] are expansion coefficients estimated via a least-square approach [31] i.e.,

$$\beta ={\left(V^{T}V\right)}^{-1}VF,$$ (8)

where

$$\begin{array}{c} V=[\begin{array}{cccccc} I & V_{\text{F}} & V_{\text{T}} & V_{\text{FF}} & V_{\text{TT}} & V_{\text{TF}} \end{array}], \end{array}$$ (9)

where *I* is an *N* × 1 unit vector (of 1s), and

$$\begin{array}{c} V_{\text{F}}=[\begin{array}{cccc} b_{1}*F & b_{2}*F & \cdots & b_{L}*F \end{array}], \end{array}$$ (10)

$$\begin{array}{c} V_{\text{T}}=[\begin{array}{cccc} b_{1}*T & b_{2}*T & \cdots & b_{L}*T \end{array}], \end{array}$$ (11)

each is a *N* × *L* matrix, while each of *V*_FF_, *V*_TT_ and *V*_TF_ is a *N* × *L*(*L* + 1)/2 matrix having columns defined by *L*(*L* + 1)/2 unique pairs of (*b*_*j*_1__*F) ⊙ (*b*_*j*_2__*F), (*b*_*j*_1__*T) ⊙ (*b*_*j*_2__*T)and(*b*_*j*_1__*F) ⊙ (*b*_*j*_2__*T), respectively, with *j*_1_ = 1,…,*L* and *j*_2_ = 1,…,*j*_1_. The notation * denotes convolution and ⊙ denotes the Hadamard product. Here *N* = *T* − 1 where *T* is the length of a time series signal.

The estimated first- and second-order self kernels of all subjects of both (ictal and interictal) groups are placed in a square matrix *Q* i.e.,

$$\begin{array}{c} \begin{array}{cc} Q & =[\begin{array}{ccccc} k_{1,1}^{n} & \sigma_{1}^{n}\times k_{2,1}^{n} & \cdots & k_{1,N}^{n} & \sigma_{1}^{n}\times k_{2,N}^{n} \end{array}\\ & \;\;\begin{array}{ccccc} k_{1,1}^{e} & \sigma_{1}^{e}\times k_{2,1}^{e} & \cdots & k_{1,N}^{e} & \sigma_{1}^{e}\times k_{2,N}^{e} \end{array}] \end{array} \end{array}$$ (12)

where $k_{1,N}^{c}$ is a first-order kernel and $k_{2,N}^{c}$ is a second-order self-kernel multiplied by the standard deviation $\left(\sigma_{N}^{c}\right)$ of the respective input for the *N*th subject for one input (T7–P7 or Fp1–F3). The superscripts n and e correspond to interictal and ictal states, respectively.

Next, a singular value decomposition is applied to matrix *Q* i.e.,

$$Q=U\lambda V^{⋆}.$$ (13)

**Fig 1:**
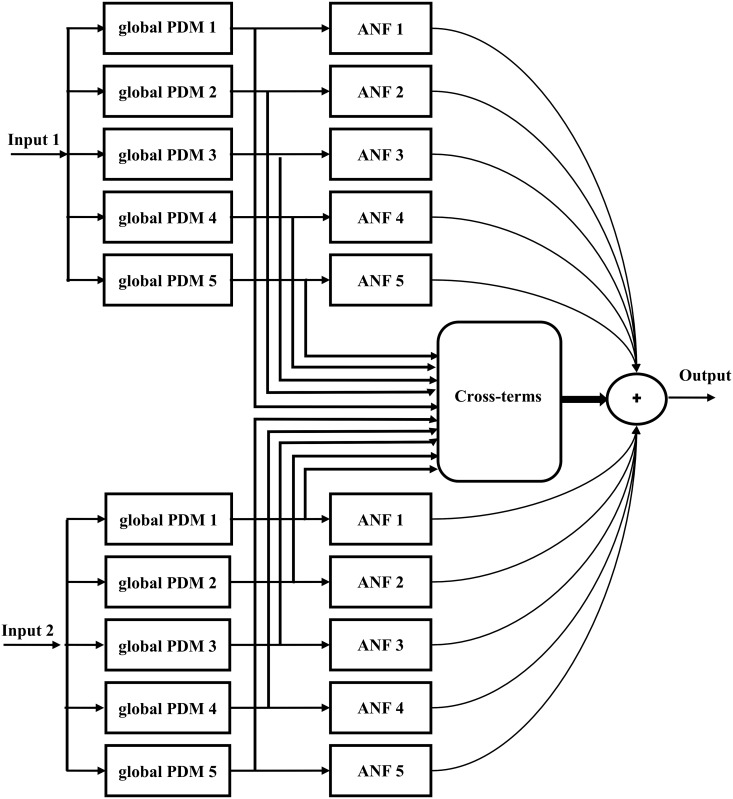
The block diagram of a dual-input global PDM model with five global PDMs. In the present study, the T7–P7 and Fp1–F3 channels are taken as input 1 and input 2, respectively, and the P3–O1 channel is considered as a model output. The ANFs are cubic polynomials. Only significant cross-terms are included in the final model. PDM, principal dynamic mode.

The columns of matrix *U* corresponding to the significant singular values (of diagonal matrix λ) form the global PDMs (i.e., *g*_*i*,T_ or *g*_*i*,F_ in Eq 14) for the respective input (T7–P7 or Fp1–F3). The output of each global PDM (by the convolution of input signal with global PDM) is passed through a nonlinear polynomial function (i.e., ANF) to capture the nonlinearity of the underlying relations. Fast Fourier transformation is used to obtain the frequency domain representation of estimated global PDMs. The intermodulatory interactions among inputs can also be determined by the cross-terms of global PDM-outputs i.e., products of ‘global PDM-output of T7–P7’ with ‘global PDM-output of Fp1–F3’. In the final model, only significant cross-terms are included, determined using the *w*-static (i.e., if the cross correlation between the cross-term and the output is greater than 99% of significance level then it is considered to be significant). Finally, the polynomial transformed outputs and significant cross-terms of global PDM-outputs are summed to form the system output i.e.,

$$\begin{array}{cc} O(n)= & \sum_{i=1}^{H}f_{i,\text{T}}\{\sum_{m_{1}=0}^{M-1}g_{i,\text{T}}\left(m_{1}\right)T\left(n-m_{1}\right)\}\\ & +\sum_{i=1}^{H}f_{i,\text{F}}\{\sum_{m_{2}=0}^{M-1}g_{i,\text{F}}\left(m_{2}\right)F\left(n-m_{2}\right)\}\\ & +\underset{\text{cross-terms}}{\underset{︸}{\sum_{m_{1}=0}^{M-1}\sum_{m_{2}=0}^{M-1}g_{i,\text{T}}\left(m_{1}\right)g_{i,\text{F}}\left(m_{2}\right)T\left(n-m_{1}\right)F\left(n-m_{2}\right)}}\\ & +\epsilon (n), \end{array}$$ (14)

where *g*_*i*,T_,*g*_*i*,F_ are the *i*th global PDMs for the T7–P7 and Fp1–F3 inputs respectively, and *H* is the number of the global PDMs accounting for both (ictal and interictal) states of all subjects. The *f*_*i*,T_ and *f*_*i*,F_ are the nonlinear functions associated with each global PDM *g*_*i*,T_,*g*_*i*,F_ respectively, termed ANFs. The general form of nonlinear polynomial is given by [31],

$$f_{i}\left(u\right)=a_{\text{1}}u+a_{\text{2}}u^{2}+a_{\text{3}}u^{3}+\cdots ,$$ (15)

where {*a*_1_, *a*_2_, *a*_3_,⋯} are polynomial coefficients, and *u* is the global PDM-output. The ANFs (defined in Eq 15) characterise the complex nonlinearities of the underlying relationships. The cross-terms (in Eq 14) contain pair products of the global PDM-outputs, and represent the interactions between T7–P7 and Fp1–F3 fluctuations. A pictorial illustration of the above described methodology is represented in Fig 1.

In this study, the optimal values of Laguerre parameter, the number of Laguerre functions, the number of global PDMs and the order of ANFs are determined using a global search procedure which minimizes the normalized mean square error across all subjects of the training data set. We found that six Laguerre functions with Laguerre parameter 0.7, and five global PDMs with cubic ANFs are optimal for both inputs on the basis of Bayesian information criteria [36] for all subjects of the training data set under both ictal and interictal states. The training data set is adopted to find the basis functions, termed global PDMs. Next, these estimated global PDMs are used to determine ANFs and their corresponding linear gain coefficients for the subjects of the test data set.

The linear gain coefficient of a global PDM is represented by the slope of a best linear line fitted to its associated cubic ANF using the least square approach. The value of the gain coefficient represents the relative strength of the associated neural rhythm i.e., the high value means that its contribution is strong while the small value suggests that its contribution is weak [37].

## 4 Statistics

All values are reported as mean±standard deviation, unless otherwise stated. The normality of the data was determined using Shapiro-Wilk test [38]. A paired *t*-test with Welch correction is performed for all comparisons to determine significant differences, with alpha defined as *p* < 0.05.

## 5 Results

**Fig 2:**
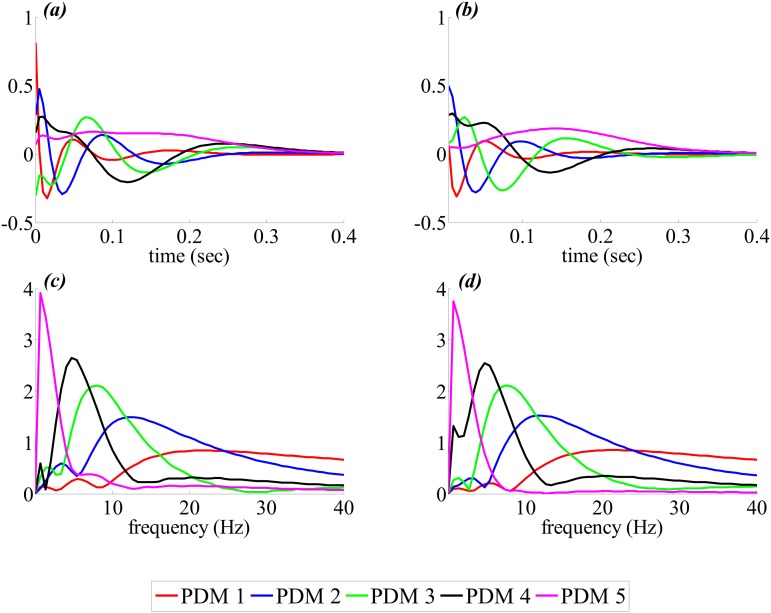
The estimated five global PDMs for the T7–P7 (left panels) and Fp1–F3 (right panels) inputs in connection with P3–O1 (as an output) in the time- (top panels) and frequency-domain (bottom panels). PDMs, principal dynamic modes.

The estimated five global PDMs from the training data set for both T7–P7 and Fp1–F3 inputs are shown in Fig 2 in the time- (upper panels) and frequency-domain (bottom panels). For the T7–P7 (Fig 2**c**), the 1st global PDM resembled high pass characteristics with relatively higher values in the beta/gamma band and a relative peak at ≈22 Hz. The 2nd, 3rd and 4th global PDMs exhibited two peaks i.e., one dominant peak and one trivial peak (Fig 2**c**). The dominant peaks were found at ≈14 Hz (high alpha band), at ≈8 Hz (high theta band) and at ≈5Hz (theta band), respectively. While the lower peaks were found at relatively smaller frequencies of ≈1-2 Hz for these three global PDMs. The 5th global PDM was found having a single peak at ≈1 Hz (delta band).

Similar to the T7–P7, the 1st global PDM for the Fp1–F3 also revealed high pass characteristics with higher values beyond ≈20 Hz (beta/gamma band) and a relative peak at ≈22 Hz (Fig 2**d**). The 2nd global PDMs were found having only one peak at ≈12 Hz (alpha band). The 4th global PDM revealed dominant peak at ≈5 Hz (theta band) with a diminishing peak at ≈2 Hz (delta band). Similarly, 3rd and 5th global PDMs demonstrated two peaks i.e., dominant peak at ≈8 Hz (high theta band) and ≈2 Hz (delta band), respectively. The relatively lower peak was found at smaller frequency of ≈3 Hz (delta) for 3rd global PDM while it was found at higher frequency of ≈5 Hz (theta band), compared to its dominant peak, for 5th global PDM (Fig 2**d**).

**Fig 3:**
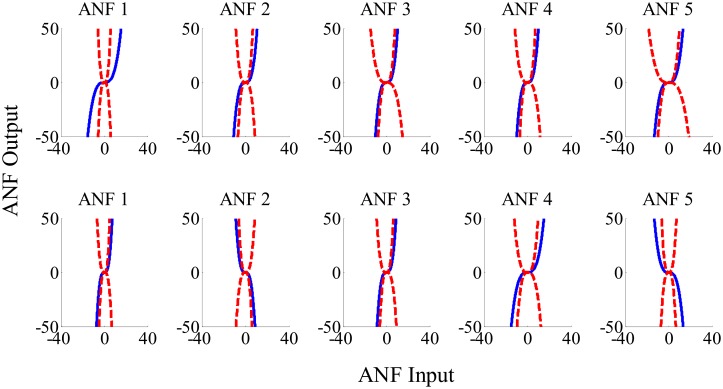
The ensemble averages of estimated cubic ANFs along with their standard deviation bounds for the T7–P7 (top panels) and Fp1–F3 (bottom panels) for the interictal states of the training data set. The solid lines represent means and dotted lines represent standard deviation bounds. Coefficients of cubic ANFs were utilized to determine the mean and standard deviation bounds. ANFs, associated nonlinear functions.

**Fig 4:**
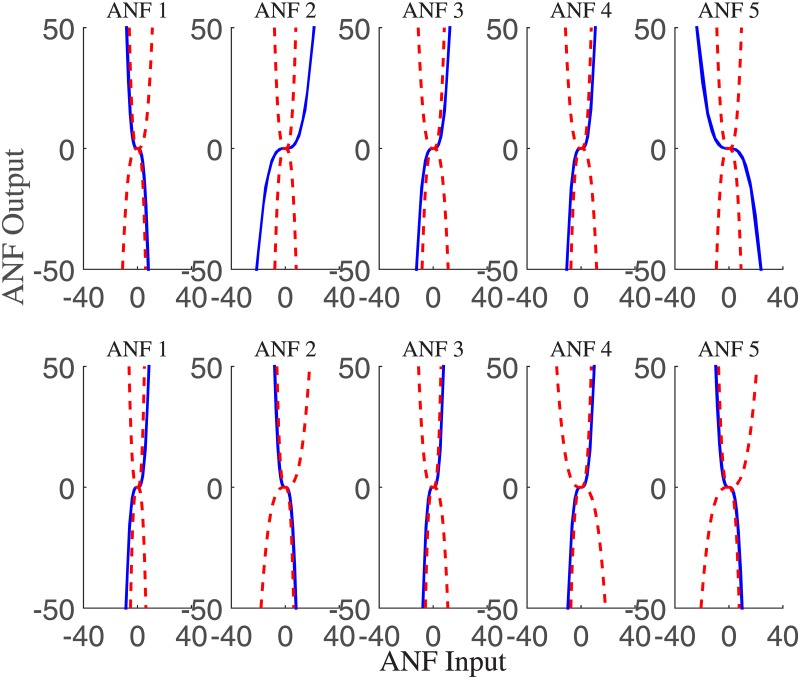
The ensemble averages of estimated cubic ANFs along with their standard deviation bounds for the T7–P7 (top panels) and Fp1–F3 (bottom panels) for the ictal states of the training data set. The solid lines represent means and dotted lines represent standard deviation bounds. Coefficients of cubic ANFs were utilized to determine the mean and standard deviation bounds. ANFs, associated nonlinear functions.

The group averaged cubic ANFs, along with the standard deviation bounds, for both T7–P7 (upper panels) and Fp1–F3 (lower panels) for the interictal state of the training data set are shown in Fig 3. We observed that ANFs associated with global PDMs for the T7–P7 (upper panels of Fig 3) had curvilinear characteristics and demonstrated high degree of variability with both positive and negative trends. Likewise, ANFs associated with Fp1–F3 global PDMs (lower panels of Fig 3) demonstrated heterogeneity with both positive and negative trends. Majority of the subjects had negative trends for ANFs associated with 2nd and 5th global PDMs. Similar curvilinear features were also observed for the group averaged cubic ANFs for both T7–P7 (upper panels) and Fp1–F3 (lower panels) for the ictal state of the training data set as shown in Fig 4. Interestingly, the 1st and 5th ANFs for T7–P7 (upper panels) of ictal state were found having opposite trends as that from interictal state. However, the trends remain similar for Fp1–F3 (lower panels) across both interictal and ictal states.

**Fig 5:**
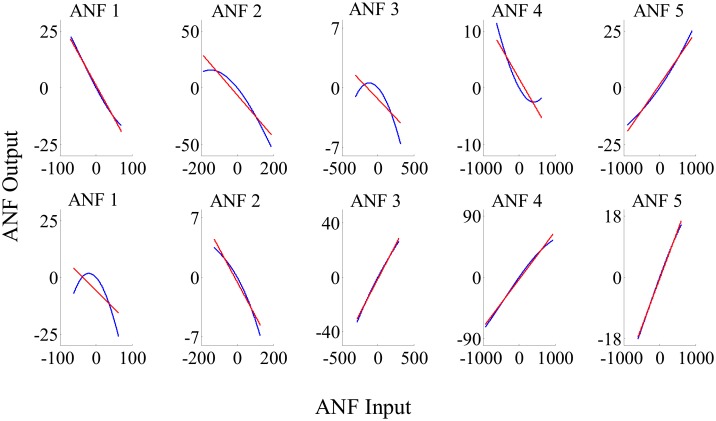
The estimated cubic ANFs for the interictal state of one representative subject of the training data set corresponding to the five global PDMs (shown in Fig 2) for the T7–P7 (upper panels) and Fp1–F3 (bottom panels), along with their best linear fits (red line). ANFs, associated nonlinear functions; PDMs, principal dynamic modes.

**Fig 6:**
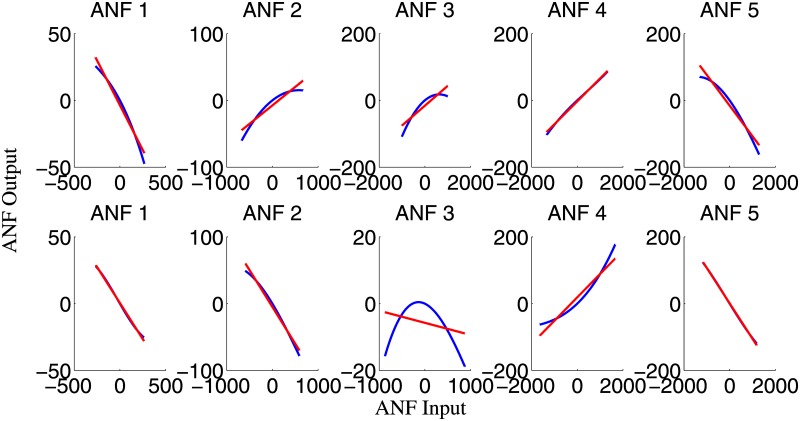
The estimated cubic ANFs for the ictal state of one representative subject of the training data set corresponding to the five global PDMs (shown in Fig 2) for the T7–P7 (upper panels) and Fp1–F3 (bottom panels), along with their best linear fits (red line). ANFs, associated nonlinear functions; PDMs, principal dynamic modes.

A representation of a linear line fitted to cubic ANFs for one subject of the training data set under interictal state is shown in Fig 5. We found that the 1st and 5th ANFs for the T7–P7 (upper panels of Fig 5) have almost linear behavior while other ANFs demonstrated curvilinear characteristics. We observed negative gain coefficients for ANFs of 1st, 2nd, 3rd and 4th global PDMs and a positive gain coefficient for ANF associated with the 5th global PDM for the T7–P7 (upper panels of Fig 5). The negative gain coefficients were found for ANFs of 1st and 2nd global PDMs of the Fp1–F3 with 1st ANF having curvilinear characteristics (bottom panels of Fig 5). The 3rd, 4th and 5th global PDMs exhibited positive gain coefficients having almost linear features (lower panels of Fig 5). Similar heterogeneous patterns were also observed across one representative subject of the training data set under the ictal state, as shown in Fig 6. The 1st and 4th ANFs for the T7–P7 (upper panels of Fig 6) and 1st, 2nd and 5th ANFs for the Fp1–F3 (bottom panels of Fig 6) have almost linear behavior while other ANFs demonstrated curvilinear characteristics. The highest degree of nonlinearity was found across 3rd ANF of the Fp1–F3. We observed negative gain coefficients for ANFs of 1st and 5th global PDMs for the T7–P7. Interestingly majority of ANFs for the Fp1–F3 demonstrated negative gain coefficients except 4th ANF which was found having positive trend.

**Fig 7:**
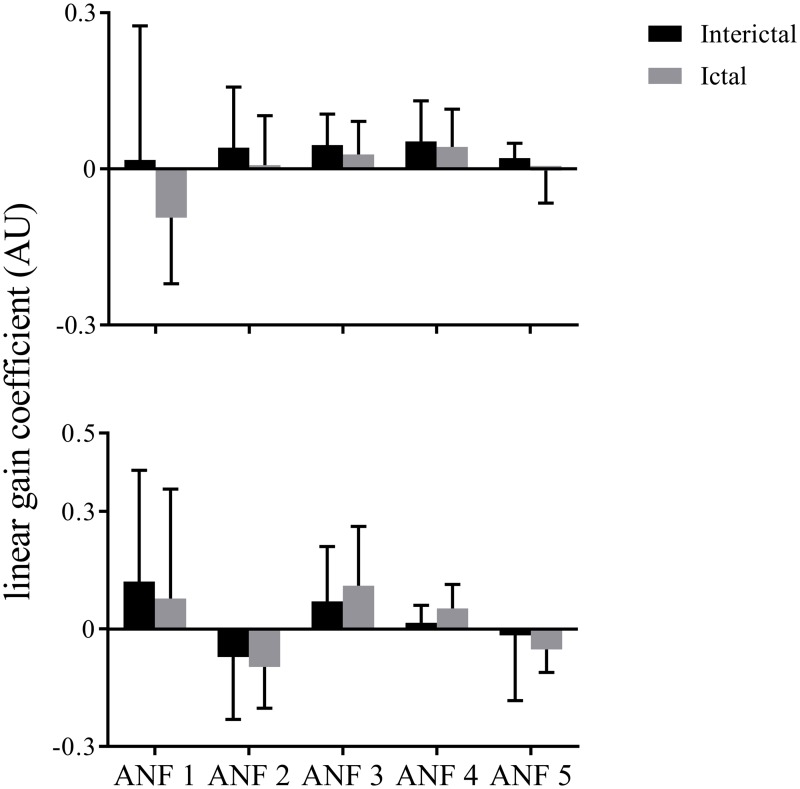
The ensemble averages of estimated linear gain coefficients (i.e., slopes of best linear lines fitted to cubic ANFs) for the T7–P7 (upper panel) and Fp1–F3 (bottom panel) for interictal and ictal states of the training data set. No significant changes were found across any ANF of either input for ictal versus interictal states of the training data set (*p* > 0.05, paired *t*-test). The error bars represent standard deviation. ANFs, associated nonlinear functions.

The ensemble averages of linear gain coefficients for the T7–P7 (upper panels) and Fp1–F3 (lower panels) under both (ictal and interictal) states of the training data are shown in Fig 7. The ensemble averages were found to be positive for all ANFs for interictal data-set for the T7–P7 (upper panels of Fig 7). However, the ictal state was found with negative mean values for 1st and 5th ANFs. The highest mean value was found across 1st ANF suggesting that its associated brain dynamics have strongest effects following epileptic seizure. Large standard deviations were observed across all ANFs for the T7–P7. The highest value was found for the 1st ANF and lowest one for the 5th ANF of the T7–P7 (upper panels of Fig 7). For the Fp1–F3, the positive ensemble means were found across 1st, 3rd and 4th ANFs while the remaining ANFs demonstrated negative means. Highest value was found for the 1st ANF and lowest value was found across the 5th ANF, suggesting that faster dynamics of the Fp1–F3 have stronger contribution while its slower rhythms have weaker effects on brain dynamics. Similar to the T7–P7, high standard deviations were found for all ANFs of the Fp1–F3, with the highest value for the 1st ANF and the lowest one for the 4th ANF.

We found no significant changes in linear gain coefficients for ictal versus interictal states of the training data set (*p* > 0.05, paired *t*-test) for any ANF of either input. This observation prompts that gain coefficients associated with a single global PDM may not differentiate between ictal and interictal states.

**Fig 8:**
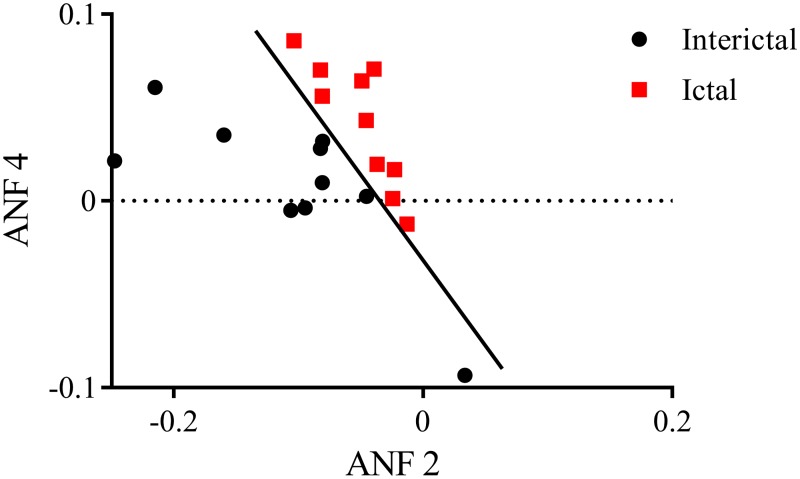
Scatter-plot of estimated linear gains coefficients of ANFs corresponding to the 2nd and 4th global PDMs for the Fp1–F3 for interictal and ictal states of the training data set. The classification line has been obtained using a linear discriminator, and shows no false-negatives and no false-positives. ANFs, associated nonlinear functions; PDMs, principal dynamic modes.

Various two-some combinations of linear gain coefficients were tested to differentiate ictal and interictal states. The present study adopted a linear discriminator for classification purposes and its accuracy was quantified in terms of false-negatives and false-positives. It is observed that clear separation between ictal and interictal states of the training data set is found using ANFs associated with the 2nd and 4th global PDMs of Fp1–F3 (as shown in Fig 8), with no false-negatives and no false-positives.

**Fig 9:**
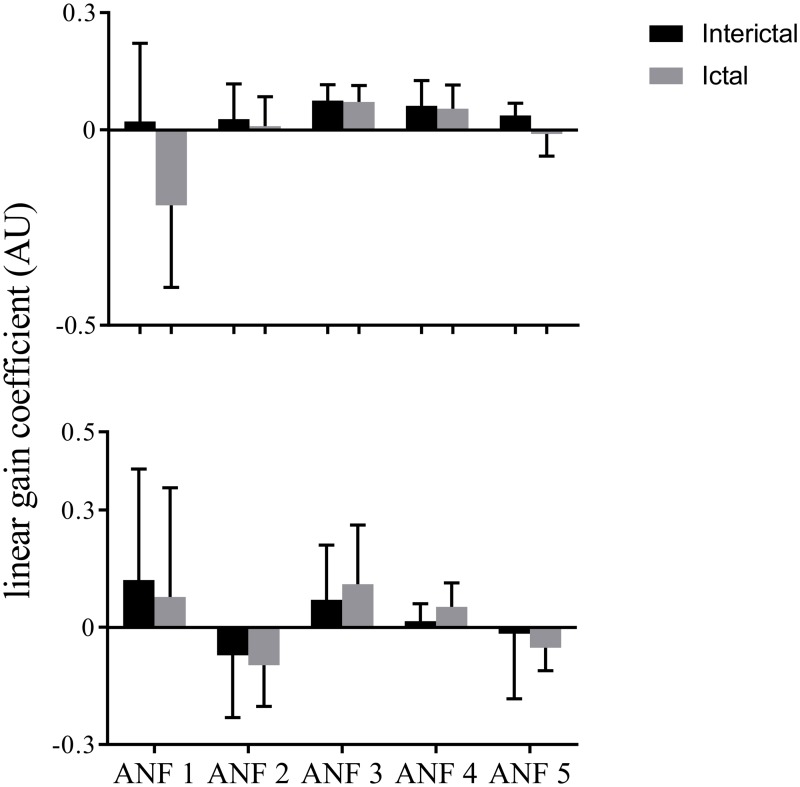
The ensemble averages of estimated linear gain coefficients (i.e., slopes of best linear lines fitted to cubic ANFs) for the T7–P7 (upper panel) and Fp1–F3 (bottom panel) for interictal and ictal states of the test data set. No significant changes were found across any ANF of either input for ictal versus interictal states of the test data set (*p* > 0.05, paired *t*-test). The error bars represent standard deviation. ANFs, associated nonlinear functions.

The predictive capability of proposed biomarkers was evaluated by examining an independent test data set i.e., global PDMs extracted from the training data set were utilized to estimate cubic ANFs for the test data set. The ensemble averages of gain coefficients corresponding to the linear lines fitted to cubic ANFs for the test data set are shown in Fig 9. Similar to the training data set, no significant changes (*p* > 0.05, paired *t*-test) were found across linear gain coefficients for ictal versus interictal states of the test data set. However, a satisfactory delineation between ictal and interictal epochs of the test data set was observed using the two-some combination of linear gain coefficients of 2nd and 4th global PDMs of Fp1–F3 channel with 8 false-negatives and 10 false-positives, achieving sensitivity of 95.7% and specificity of 95.2%. This observation shows that ANFs associated with the 2nd and 4th global PDMs of Fp1–F3 can be utilized as functional biomarkers for characterisation of ictal and interictal states of epilepsy.

## 6 Discussion

### 6.1 Main findings

The present study characterises the changes in functional coupling of the temporal and frontal lobes with the occipital segment of a human brain during ictal and interictal episodes. To achieve this, a relatively novel approach of global PDMs is utilized to quantify the complex causal dynamics of EEG (with T7–P7 and Fp1–F3 channels as inputs and P3–O1 channel as an output). Keeping with our hypothesis, we found that the linear trends of ANFs associated with the 2nd and the 4th global PDM (representing delta, theta and alpha bands) of Fp1–F3 may distinguish ictal and interictal states of epilepsy. These findings provide an external support to the observations of a recent study [25], examining simulated epileptic EEG data, that nonlinearities linked to global PDMs of distinct spectral features may have potential to characterise epileptic seizure dynamics.

### 6.2 Modeling of epileptic seizures

Over the past few years, EEG has gained tremendous success as an essential tool for better understanding of the human brain function [39] because it can directly measure the electrical brain activity, as the primary effect [39, 40]. It also offers superior temporal resolution of few milliseconds [40]. The evolution of seizures in newborns causes the EEG to transit between ictal and interictal states in a non-stationary fashion [12]. These distinctive features prompt the utilization of advanced analytical approaches to examine EEG for the detection and quantification of epileptic seizures. To this end, Liu et al. [41] adopted the autocorrelation function to distinguish the rhythmicity of electrographic seizure from the normal background cerebral activity. In a follow up, Gotman et al. [42, 43] performed a series of experiments and evaluated spectral characteristics of rhythmic paroxysmal discharges across a wide range of frequencies, along with their repetitive spike patterns, and observed that almost always seizures are across the 3–29 Hz range. Kitayama et al. [44] performed nonstationary analysis of EEG using wavelet transform to recognize and characterise the frequency profiles of neonatal seizures, and observed these seizures in the range of 0.5–13 Hz with dominant impact across 0.5–6 Hz. In another study, Green et al. [45] examined a number of features, extracted using frequency-domain, time-domain and entropy-based techniques, to discriminate between seizure and non-seizure epochs across three frequency ranges of 0.5–20 Hz, 0.5–32 Hz and 2–20 Hz. The findings of this study show that 2–20 Hz provides the best discriminatory power to distinguish seizure from that of non-seizure epochs.

Epilepsy being a network disorder can disrupt both the functional and effective connectivity of the brain segments [18]. To date, a variety of promising techniques have been proposed to quantify the brain segregation and integration, including model-based and data-driven techniques for effective connectivity, and linear, nonlinear and information-based techniques for functional connectivity. These schemes have been comprehensively reviewed by Sakkalis in [46]. Sakkalis et al. [47] proposed linear and nonlinear synchronization measures to characterise children epilepsy, and observed a significant disruption in the functional coupling of EEG channels in terms of the reduced phase synchronization. Similarly, Adebimpe et al. [48] observed the reduced functional connectivity, especially in alpha and beta bands, by employing the lagged phase synchronization. Coben et al. [20] employed Granger causality estimates to analyze effective connectivity of children EEG and reported hypercoupling near the seizure foci and low causality measures across nearby and associated neural pathways. These findings were also found to be consistent with the other studies applying a dynamic causal modeling approach [49].

Recently, V. Z. Marmarelis [33, 34] suggested the use of nonlinear moving average models in connection with Volterra kernels to examine the complex causal coupling of different time series signals. These linear and nonlinear kernels and their derived global PDMs have been employed for modeling of various physiological systems [50, 51] including EEG dynamics [31]. For example, Song et al. [52] studied the short term plasticity of synapses in the central nervous system using Volterra kernels. Kang et al. [31] employed the concept of global PDMs and evaluated the characteristics of their associated ANFs to differentiate Alzheimer’s patients from healthy individuals using EEG.

In the context of epileptic seizure dynamics, only one study [25] has employed the notion of global PDMs, but to the simulated EEG data, and found that the ANFs of two global PDMs having sustained spikes at ≈25 Hz (beta band) and ≈40 Hz (gamma band) may identify the epileptic seizure activity. The present study is an attempt to externally validate the proposal of classification indices suggested by Cao et al. [25] by examining a clinical EEG data set having the presence of ictal and interictal states of epilepsy. We found that the linear gain coefficients of subject-specific ANFs associated with the 2nd and 4th global PDM of Fp1–F3 (representing delta, theta and alpha bands) may be adopted as markers of ictal and interictal states of epilepsy. These observations imply that ictal state affects the functional connectivity of temporal and frontal lobes with the occipital brain segment across delta, theta and alpha neural rhythms, and are found to be consistent with the previous studies [44, 45, 48, 53–55] exhibiting epileptic seizure related changes across < 20 Hz frequency range. For example, Schmidt et al. [53] found that, in contrast to healthy group, the synchronization of functional networks is significantly decreased for the epilepsy cohort across both theta (3–6Hz) and low-alpha (6–9 Hz) bands. Similarly, Adebimpe et al. [48] also observed that the functional connectivity of brain networks in epileptic patients is associated with higher phase synchronisation values across theta and alpha neural rhythms. Further, recent studies [54, 55] demonstrated temporal lobe epilepsy affecting the delta neural rhythms. To summarize, the findings of the present study are in agreement with the existing literature.

### 6.3 Pathophysiological interpretation and implications of global PDMs

Generally, the pathophysiological origins of global PDMs are not clear, however following plausible explanations are worth considering. The 1st global PDMs of both T7–P7 and Fp1–F3 demonstrate high pass characteristics with dominant peaks at ≈20 Hz and ≈22 Hz, respectively. The higher values are observed across/beyond beta/gamma band for both global PDMs. This indicates that transient fluctuations, slower than the beta/gamma band, from (T7–P7 channel of) left temporal and (Fp1–F3 channel of) frontal lobe to (P3–O1 channel of) occipital lobe of the brain are more effectively attenuated. The 2nd global PDM of T7–P7 has two spectral peaks which might be affected by the functional coupling between higher alpha and higher delta neural rhythms. Similarly, the 3rd and 4th global PDMs of T7–P7 also have two spectral peaks which might be related to the functional synchrony between delta and theta neural rhythms. The 5th global PDM has only a single peak which might be associated with delta neural activities.

The presence of single peak for 2nd global PDM of Fp1–F3 demonstrates that neural rhythms across alpha band might be sensitive to these global PDMs. The 3rd and 4th global PDM exhibits functional association of delta bands with theta bands. The 5th global PDM of Fp1–F3 also exhibits two spectral peaks which might be sensitive to the neural rhythms of delta and theta bands. In contrast to other dual-peak global PDMs, the dominant peak for 5th global PDM of Fp1–F3 prevailed at a smaller frequency while the relative lower peak appeared at higher frequency value. It is worth mentioning that the viable hypothesis of dual-peak global PDMs in terms of neural synchrony between different neural rhythms presented in the present study still remains to be externally validated and verified. However, our findings reveal that epilepsy affects the functional coupling of the 2nd and 4th global PDM of Fp1–F3 (delta, theta and alpha bands) in response to P3–O1 dynamics of EEG time series. Moreover, these complex featured dual peak global PDMs might also alter with neural impairment during stroke, dementia, autism and Parkinson’s disease.

The global PDMs associated with delta band might be sensitive to short term plasticity, prospect of reaction and neuropsychological conduct [56]. The ‘alpha band’-related global PDMs might be susceptible to internalized attention and mental activity [56]. Similarly, the ‘beta neural rhythm’-related global PDMs might get affected by changes in sensorimotor integration and motor action [56]. The global PDMs associated with theta band might be susceptible to memory tasks, such as memory formation, behaviour and cortical synchronization [56].

Interestingly, the associations of 1st, 2nd and 3rd global PDMs of both inputs are found across common neural rhythms. This data supports the strong connection between the temporal and frontal lobes with the occipital lobes. However, the absence of significant cross-terms across these (temporal and frontal) brain segments indicates that they might be contributing independently.

The curvilinear ANFs highlight the presence of complex nonlinearities embedded in brain dynamics. Interestingly, all ANFs are found to be symmetric across the origin, suggesting that a similar behavior in the output is found across positive and negative input fluctuations. A wide range of positive and negative trends of ANFs might suggest that intensities of pathophysiological mechanisms contributing to brain dynamics across distinct time-scales are diverse and individual-dependent. These findings are consistent with the heterogeneous behavior of other cerebral dynamics e.g., autoregulation of cerebral blood flow [57]. Positive trends of ANFs suggest the proportional cause of input fluctuations to the output dynamics i.e., an increase in the input triggers the output to rise and vice versa. While, the negative trends represent a reciprocal relation between the input and the output. One plausible explanation for the negative trend might be the existence of a feedback mechanism i.e., an increase in output may instruct a drop in the input for the cerebral homeostasis. The linear trends of cubic ANFs demonstrate that both, very slow (low-pass global PDM) and very fast (high-pass global PDM), fluctuations of the T7–P7 are linearly buffered while other rhythms are attenuated in a complex fashion by the P3–O1. In contrast, all frequency components of the Fp1–F3 are linearly buffered except very high frequency neural rhythms (associated with high-pass global PDM) which are reflected in a nonlinear non-symmetric fashion by the P3–O1.

The presence of high variations across linear gain coefficients might prompt that faster brain dynamics (high pass global PDM) associated with the T7–P7 demonstrate a high degree of heterogeneity while very slow oscillations (low-pass global PDM) reveal reduced diversity as compared to other cerebral fluctuations (associated with 2nd, 3rd and 4th global PDMs). Similarly, high frequency rhythms of the Fp1–F3 might have diverse effects on brain fluctuations, and differ significantly across individuals.

The findings of the present study in terms of biomarkers extracted from different EEG channels across different neural bands may have important methodological implications. First, the application of two different EEG channels implicates that the complex disease of epilepsy should be characterised by examining the functional connectivity of different brain segments [58, 59] using advanced signal processing tools. Moreover, the evidence of different neural bands (delta, alpha and theta) associated with epileptic seizure suggests the use of filter bank approaches to focus on a wide array of adaptive frequency ranges instead of a single wider spectra.

It is also worth emphasizing that 4 out 5 global PDMs found in Alzheimer’s disease study [31] are strikingly similar to those of the present epilepsy study. This observation points to an important pathophysiological implication that the basis functions (termed global PDMs) remain appreciably similar across different brain diseases while trends of their associated nonlinear functions might differ across diseases.

### 6.4 Pathophysiology of epilepsy

Epilepsy being a dynamical disease exhibit characteristics of neural networks with the presence of at least two states, named ictal and interictal activities [60]. The evolution from one state to the other for such kind of system can be either gradual or abrupt [61]. An endogenous or external stimuli slowly deforms the system attractor during the gradual transition. While the abrupt transition is stimulated by an endogenous factor or a random perturbation, and is manifested by the simultaneous presence of ictal and interictal attractors [61]. The seizure may be predicted in its early phases during gradual transition while its detection during convoluted abrupt changes may not be evident from the traditional EEG dynamics, and may require estimating the system response to an externally applied stimuli [60] [61].

Toward this, Taylor et al. [62] proposed a single-pulse stimulation for the premature termination of spike-wave seizure in the epileptic EEG by utilizing a neural population model. Assuming bistability of the epilepsy, the proposed method employs a state space structure in terms of a basin of attraction to quantify the background EEG activity. The basin structure is found to be within certain limits under normal brain functioning while the epileptic activity causes prominent deviations in basin paths. The application of single-pulse perturbation ensures the brain activity return to the state within the basin. This study utilized the proposed stimulation approach to abate noise-free and noise-induced spike-wave seizures successfully.

Recently, Fan et al. [63] proposed a neural field model to uncover the mechanisms of neural behavior, and investigated the pathology of epilepsy. It was observed that the disinhibitory action of two selected inhibitory variables of the proposed model resulted in stable tonic-clonic oscillations and periodic spikes with slow-wave discharges (i.e., absence seizure). The manifestation of disinhibition could be responsible for the transition of tonic-clonic seizure to absence seizure, e.g., decrease in the inputs of gamma-Aminobutyric acid (GABA_A_) receptor can induce the occurrence of spike-wave behavior.

In a recent follow-up study, Wang et al. [64] employed a mean-field modeling approach and found that the slow kinetics of GABA_B_ receptors on thalamus reticular nucleus (TRN) lead to spike-wave discharges (SWDs). The deep brain stimulation (DBS) was applied to the TRN which helped in eliminating the SWDs.

To sum, the stand-alone application of traditional or modern modeling approaches may not be sufficient to apprehend the complex multistable dynamical phenomenon of epilepsy. The observations of the above mentioned literature suggest that certain stimulation techniques should be adopted along with the modeling approaches for the efficient abatement of epileptic seizures, specifically, in children epilepsy.

### 6.5 Methodological considerations

Several methodological limitations need to be considered before the interpretation of findings of the present study. First, the proposed biomarkers could be inevitably sensitive to the selection of input/output channels for the global PDM analysis. In the current study these channels (T7–P7 and Fp1–F3 as inputs and P3–O1 as an output) are arbitrarily selected based on the following observations: 1) a recent study by Chang et al. [29] examined the same dataset (as of the present study) and reported that the selection of left/right temporal lobes (T7/T8) and extrafocal (O1) channels provides the same classification accuracy as the 22-channel case. These observations led us to the selection of the left temporal (T7-P7) and extrafocal (P3-O1) channels. The remaining third channel i.e., Fp1-F3 was selected based on the method adopted by Bhattacharyya et al. [30] i.e., quantitative measure of mutual information was used to find a channel having highest interdependency to the T7–P7, 2) the volume conduction across the nearby channels may generate spurious results [31], and 3) these channels have the minimal presence of the ocular activity [31]. However, future studies can utilize the notion of information flow, e.g., Granger causality analysis [20], to determine cause and effect channels. Subsequently, cause and effect channels can be adopted as input and output, respectively, to characterise the underlying system dynamics. Moreover, the comorbidity generated by epileptic seizures has high degree of inter-subject variability for specific types of seizures, resulting in varying changes across different EEG channels [65]. Therefore the fixed channel selection strategy for all subjects might generate biased results, and need further investigation. One approach in this direction might be to reduce the redundancy of the information embedded in all nearby channels of a specific brain segment using, for example, principal component analysis (PCA) [65, 66] which extracts principal components from all nearby channels of a specific region. The application of PCA also ensures the removal of electrooculogram and thereby improves the signal-to-noise ratio [65, 66].

Second, we recognize that our sample size is not large enough to suggest the application of proposed biomarkers in clinical diagnosis. Further that the adopted data sample includes a diversity of patients (males, females) of varying ages (1.5–22 years) with the presence of different types of seizures (clonic, atonic and tonic). With the objective of automatic seizure detection, recent studies [26, 28, 30] have suggested modern signal processing and machine learning tools which consider the whole pool of (young children and adults) patients of the same dataset (as of the present study). The present study is also an attempt to propose new indices using the unique and novel framework of global PDMs which provides a common reference frame to compare various pathophysiological states. This compact formulation has been quite successful in distinguishing healthy and Alzheimer’s patients [31], normal and impaired cerebral autoregulation [50], and has been reported with the reduced inter-subject variability [67]. Though results of the present study are also quite promising, yet the sex- and age- and seizure-type-related differences associated with the connectivity of brain regions remain unexplored, and need further investigation in future studies.

Third, the present study analyzed only short epochs of EEG for model training. We acknowledge that longer duration of time series can result in a reduced variance of the estimated model parameters. However, the EEG signals examined in the present study are of comparable duration to that of a previous study [31] employing the same approach (of global PDMs) for diagnosis of Alzheimer’s disease.

Fourth, although the preliminary results of the present study suggest that EEG can promise standalone to distinguish between normal and epileptic activity, other imaging techniques [68] should also be adopted as complementary tools for the visual inspection of brain activities to get improved and robust detection of epilepsy onset.

Finally, it is also worth emphasizing that the present study focused on the general intractable epileptic seizure activity in the presence of various types of seizures (clonic, atonic and tonic). Authors warrant the extrapolation of the proposed biomarkers to the clinical setup, and suggest further investigation. For example, future studies should focus on the specific types of epileptic seizures to explore their associations with the global PDMs and the trends of their associated ANFs, and the connectivity across different brain regions.

## 7 Conclusion

In summary, the delta, theta and alpha neural rhythms might be associated with the epileptic activity. The observations of the present study also suggest that global PDMs and their associated ANFs may offer potential utility as functional biomarkers to highlight the brain dynamics.

## Supporting information

S1 File Seizure details of all subjects. (XLSX)

S2 File Training and test data sets. (DOCX)
