# Supplementary material for: Characterisation of ictal and interictal states of epilepsy: A system dynamic approach of principal dynamic modes analysis
Source: PLoS One. 2018 Jan 19;13(1):e0191392. doi: 10.1371/journal.pone.0191392 (PMC5774786; doi:10.1371/journal.pone.0191392)
Supplement: S2 File — (DOCX) [file pone.0191392.s002.docx]

**S1 File:** Description of the training and testing data sets.

**Training Data set:** The initial 69-sec epochs of one ictal and one interictal state (labeled as training data in excel file ) from the following 10 subjects were used as training dataset.

Chb03, Chb04, Chb09, Chb10, Chb11, Chb15, Chb18, Chb19, Chb23, Chb24.

These subjects were randomly selected. One of these 10 subjects (i.e., Chb18) has a longest ictal activity of only 69 seconds. Since the adopted PDM approach requires data to be of equal length for all subjects, initial 69-sec records from all 10 subjects of the training dataset were utilized for model training.

**Test data set:** The remaining 188 seizures of all durations of all subjects were included in the test dataset.
